# DIAPH3 is a multifaceted prognostic biomarker that links immunotherapy response to tumor microenvironment in prostate cancer

**DOI:** 10.1007/s12672-026-04413-6

**Published:** 2026-01-16

**Authors:** Yuxuan Chen, Ping Wang, Shuping Yang, Gang Jia, Lei Jia, Rui Zhu

**Affiliations:** 1 Department of Urology, People’s Hospital of Ningxia HuiAutonomous Region, Yinchuan, 750000 Ningxia HuiAutonomous Region China; 2 Department of Pathology, People’s Hospital of Ningxia HuiAutonomous Region, Yinchuan, 750000 Ningxia HuiAutonomous Region China; 3 Department of Nursing, People’s Hospital of Ningxia HuiAutonomous Region, Yinchuan, 750000 Ningxia HuiAutonomous Region China

**Keywords:** DIAPH3, Prognosis, Prostate cancer, Radiotherapy, Immunotherapy

## Abstract

**Objective:**

To systematically investigate the prognostic value of DIAPH3 in prostate cancer (PCa) and explore its potential role in linking immunotherapy response and tumor microenvironment remodeling.

**Methods:**

We analyzed TCGA-PRAD, Stockholm, Moffitt, UK, and Yinchuan cohorts. DIAPH3 expression was compared between tumor and adjacent tissues. Kaplan–Meier and multivariate Cox analyses were conducted to assess BCRFS and PFS. Immunotherapy response was analyzed using TIDE and IPS. Gene expression, somatic mutations, and immune microenvironment status were analyzed.

**Results:**

High DIAPH3 expression correlates with poor prognosis and is an independent predictor of BCRFS, PFS, and RFS. Functional analyses show its involvement in cell cycle regulation and IL-17 signaling. Elevated DIAPH3 correlates with altered immune infiltration, including increased Th2 lymphocytes and decreased NK cells and pDCs. DIAPH3 expression associates with immunotherapy response, validated in a pan-cancer cohort. Drug sensitivity analysis revealed PI3K inhibitors are more effective in low DIAPH3 tumors, while PARP inhibitors are more effective in high DIAPH3 tumors.

**Conclusion:**

DIAPH3 is a robust prognostic biomarker in PCa, linked to poor prognosis, immune infiltration, and therapeutic response. High DIAPH3 expression correlates with aggressive tumor progression and poor survival outcomes, highlighting its value for prognostic modeling and therapy selection.

**Supplementary Information:**

The online version contains supplementary material available at 10.1007/s12672-026-04413-6.

## Introduction

Prostate cancer (PCa) is the most common malignancies of the genitourinary system worldwide and constitutes a leading cause of cancer-related mortality [1]. Despite the availability of several treatment options for PCa, nearly half of the patients encounter disease recurrence, leading to an unfavorable prognosis [2]. Therefore, the surveillance of disease recurrence represents a critical strategy to improve the prognosis of PCa patients. Prostate-specific antigen (PSA) is typically used to detect and monitor recurrence in PCa patients [3, 4]. In addition, tumor stage and Gleason score have been identified as risk factors for progression-free survival (PFS) in PCa [5]. However, current biomarkers remain inadequate to comprehensively predict the recurrence of PCa.

Diaphanous-related formin 3 (DIAPH3), a member of the formin family, serves as a major effector of Rho GTPases [6]. Previous studies have demonstrated its involvement in cell division and morphological differentiation [7, 8]. This gene is implicated in cancer cell migration and metastasis, including in pancreatic cancer [9]. Interestingly, the prognostic effects of DIAPH3 appears to differ across various types of cancers, as it has been reported to inhibit tumor progression in breast and colon cancers [10, 11]. The role of DIAPH3 in the prognosis of PCa remains unclear; however, some evidence suggests that DIAPH3 deficiency may be correlated to the transition of PCa cells to an amoeboid tumor phenotype [12]. There is also evidence that DIAPH3 expression may be associated with immune cell infiltration and immune checkpoint expression [13, 14]. Thus, DIAPH3 may regulate the recurrence of PCa by remodeling the tumor microenvironment. Nevertheless, the immune characteristics associated with DIAPH3 in PCa require further elucidation.

Therefore, this study was designed to systematically investigate the expression of DIAPH3 and its association with biochemical recurrence-free survival (BCRFS) and progression-free survival (PFS) in PCa patients, as well as to investigate the potential underlying mechanisms. The significance of this study lies in its potential to advance our understanding of DIAPH3-mediated processes in the progression of PCa, which may facilitate the identification of novel biomarkers and therapeutic targets to improve patient management and clinical outcomes.

## Materials and methods

### Data download and clinical sample collection of PCa

The transcript, clinical and somatic mutation information of TCGA-PRAD patients were downloaded from TCGA GDC portal (https://gdc.cancer.gov/), and data download were conducted by R package TCGAbiolinks. All patients were included due to completed prognostic information, such as BCRFS and PFS time. Gene expression, clinical features and follow-up time (BCRFS) of CamCap cohort (GSE70768), Stockholm cohort (GSE70769) and Moffitt cohort (GSE54460) were obtained from Gene Expression Omnibus (GEO) database [15–21]. The paraffin sections of 37 PCa patients (adjacent tissues were obtained from nine of these patients) in People’s Hospital of Ningxia HuiAutonomous Region between 2019 and 2023 were collected. All experimental protocols were approved by Ethics Committee of Ningxia Hui Autonomous Region People’s Hospital (approved ID: [2021]-NZR-127). Patients of these cohorts received radical prostatectomy (RP). The UK cohort (GSE116918) was also obtained from GEO database, in which patients received radical radiotherapy (RRT).

### Immunohistochemical analysis

Immunohistochemical staining was performed on formalin-fixed, paraffin-embedded sections using the following protocol: Tissue sections were dewaxed in xylene (3 × 10 min) and rehydrated through a graded ethanol series. Antigen retrieval was conducted by heating slides in citrate buffer (10 mM, pH 6.0) at 95°C for 20 min. Endogenous peroxidase activity was blocked with 3% H₂O₂ in methanol for 15 min at room temperature. After blocking with 5% normal goat serum for 1 h, sections were incubated with primary antibody against DIAPH3 (rabbit polyclonal, 1:300 dilution; Proteintech, Cat#14342-1-AP) at 4 °C overnight. Slides were then treated with horseradish peroxidase (HRP)-conjugated goat anti-rabbit IgG polymer (ready-to-use; EnVision + System, Dako) for 50 min at room temperature. Signal development was achieved using 3,3’-diaminobenzidine (DAB) substrate (Dako) with microscopic monitoring (typically 1–3 min). Sections were counterstained with Mayer’s hematoxylin for 1 min, differentiated in 1% acid alcohol, blued in 0.2% ammonia water, dehydrated, and mounted with resinous medium. Negative controls omitting the primary antibody showed no specific immunoreactivity.

### Survival analysis for PCa patients

BCRFS is BCR free survival, and the defined event is: biochemical relapse was defined according to European Guidelines as a persistent rise above 0.2 ng/ml [22]. PFS is the period from the date of diagnosis until the date of the first occurrence of a new tumor event (NTE), which includes progression of the disease, locoregional recurrence, distant metastasis, new primary tumor, or death with tumor [16]. In addition, BCRFS and PFS of PCa patients were evaluated in our study, and Kaplan–meier (KM) curves was firstly performed to analyze the PFS difference between low and high DIAPH3 expression groups that were divided by R package survminer based on log-rank test. To reduce the bias of survival analysis, the clinical characteristics, including age, PSA, TNM stage, Gleason score and surgical margin status were further adjusted in Cox multivariate regression analysis. In survival analysis, two-side *P*-value < 0.05 was considered as significant.

### Concepts of radical radiotherapy

Radical radiotherapy is an adjunctive treatment without surgery. Some patients with prostate cancer who are intolerant to surgery or refuse surgery can choose to directly undergo radiotherapy without surgery. This concept contrasts with “palliative care”, which aims mainly at alleviating symptoms and prolonging survival.

### Development and validation of nomogram

To develop a nomogram model for predicting the PFS in PCa patients, stepwise regression based on AIC value minimum was performed to screen variables. The clinical data were recoded as follows: age was categorized as < 60 and ≥ 60 years old, and PSA value was categorized as < 4, 4–10 and > 10 ng/ml. Gleason score was categorized as ≤ 6, 7, 8 and 9–10. For BCRFS, patients from TCGA cohort were utilized to develop model and CamCap, Stockholm and Moffitt cohorts were applied for validation. For PFS, the patients from TCGA cohort were utilized to develop model and Yinchuan cohort was applied for external validation. The model with lowest AIC value was constructed by R package regplot. Accuracy and calibration of nomogram model was evaluated by C-index value and calibration curves. The 95% CI of C-index and comparsion between model with and without DIAPH3 were analyzed by R package CsChange.

### Differential expressed gene and gene function enrichment analysis

Expression profiles of DIAPH3 in tumor and adjacent tissues was compared in paired and unpaired samples from TCGA and Yinchuan cohorts, and wilcoxon test was applied to evaluated the difference. Meanwhile, differentially expressed genes between high DIAPH3 expression group and low DIAPH3 expression group were analyzed using R package Deseq2. The optimal cut-off value in survival analysis of PFS was used for dividing the groups with high and low expression of DIAPH3. When gene expression was 0 in all samples, it would be excluded during the analysis. For differentially expressed genes between groups, the *P*-value was corrected by Benjamini-Hochberg (BH) method to obtain false discovery rate (FDR). Genes were considered as significantly differentially expressed whose FDR < 0.05 and |log2 (fold change) |> 1. In order to analyze the potential biological processes of differentially expressed genes between high and low DIAPH3 expression groups, gene function enrichment analysis was performed. The Kyoto Encyclopedia of Genes and Genomes (KEGG) gene set was referenced. The *P*-value was adjusted by BH method, and adjusted *P*-value < 0.05 was considered as enriched in the analysis.

### Tumor somatic mutation and tumor immune microenvironment analysis

Tumor somatic mutations are crucial to the prognosis and treatment of cancer patients. In this study, the association between DIAPH3 expression and gene somatic mutations was analyzed in samples of PCa patients. For tumor somatic mutant genes, only non-synonymous mutations were considered in this study. Frame_Shift_Del, Frame_Shift_Ins, Missense_Mutation, Nonsense_Mutation, Splice_Site, In_Frame_Del, In_Frame_Ins, Translation_Start_Site, and Nonstop_Mutation were included as non-synonymous mutations. For each gene, it was coded as mutated and wild, and the gene with mutation rate greater than 2% was compared between DIAPH3 groups using permutation test, and the *P*-value < 0.01 was considered to be significantly and differentially mutated genes.

For immune microenvironment analysis, 24 immune cell infiltration levels were calculated by single-sample gene set enrichment analysis (ssGSEA) method, and scores of 7 kinds of immune signature gene sets were calculated by geometric mean method, including immunosuppression signature (gene set: CXCL12, TGFB1, TGFB3, LGALS1), T cell activation signature (gene set: CXCL9, CXCL10, CXCL16, IFNG, IL15), T cell survival signature (gene set: CD70, CD27), Regulatory T cell signature (gene set: FOXP3, TNFRSF18), MHC signature (gene set: HLA-A, HLA-B, HLA-E, HLA-F, HLA-G, B2M) Myeloid signature (gene set: CCL2) and TLS signature (gene set: CXCL13). The difference of immune checkpoints, including PDCD1, CD274, PDCD1LG2, CTLA4, HAVCR2, LAG3, CD276, CD247, TNFRSF9, TNFRSF4, TLR9, between two groups was compared by Wilcoxon test, and the *P*-value was corrected by BH method to obtain FDR value, FDR < 0.05 was considered statistically significant.

### Evaluation of immunotherapy sensitivity of PCa patients

Immunophenoscore (IPS) and Tumor Immune Dysfunction and Exclusion (TIDE) algorithms were performed to evaluate the PCa patient’s response to immunotherapy. Generally, high IPS and lower TIDE score could predict better immunotherapy response. Additionally, an integrate pan-cancer immunotherapy cohort includes bladder cancer, esophageal adenocarcinoma, glioblastoma, melanoma and urothelial cancers. The survival analysis was performed in KM plotter website (https://kmplot.com/analysis). Based on IPS and TIDE scores, we performed mediation analysis for tumor immune microenvironment. DIAPH3 expression was regarded as exposure, and IPS and TIDE scores were regarded as outcome representing immunotherapy response. 24 immune cells calculated by ssGSEA method were analyzed as potential mediators using R package mmabig.

### Potential drug intervention for DIAPH3 in PCa patients

The sensitivity value (IC50) of 213 drugs for PCa patients in TCGA cohort was predicted by Oncopredict algorithm based on transcriptomic data of PCa cell lines, including VCaP, DU-145, PWR-1E, PC-3, LNCaP-Clone-FGC and 22RV1 cells in genomics of drug sensitivity in cancer (GDSC, https://www.cancerrxgene.org/) database. Moreover, the drug sensitivity data of PCa cell lines in CTRP 2.0 (https://portals.broadinstitute.org/ctrp.v2.1/) and PRISM (https://www.theprismlab.org) was obtained to explore the potential drugs in treating patients with high/low DIAPH3 expression. The area under the dose–response curve (area under the curve, AUC) values in two databases were used for the measurement of drug sensitivity, and lower AUC values indicate increased sensitivity to treatment. During the identification of drug intervention in the two databases, we required that correlation coefficient between DIAPH3 expression and drug sensitivity should be more than 0.5. The sensitivity difference of drugs between low and high DIAPH3 expression groups was evaluated by wilcoxon test, in which *P* value was adjusted by BH method to be less than 0.0001.

## Results

### The expression and prognostic value of DIAPH3 in PCa

**Fig. 1:**
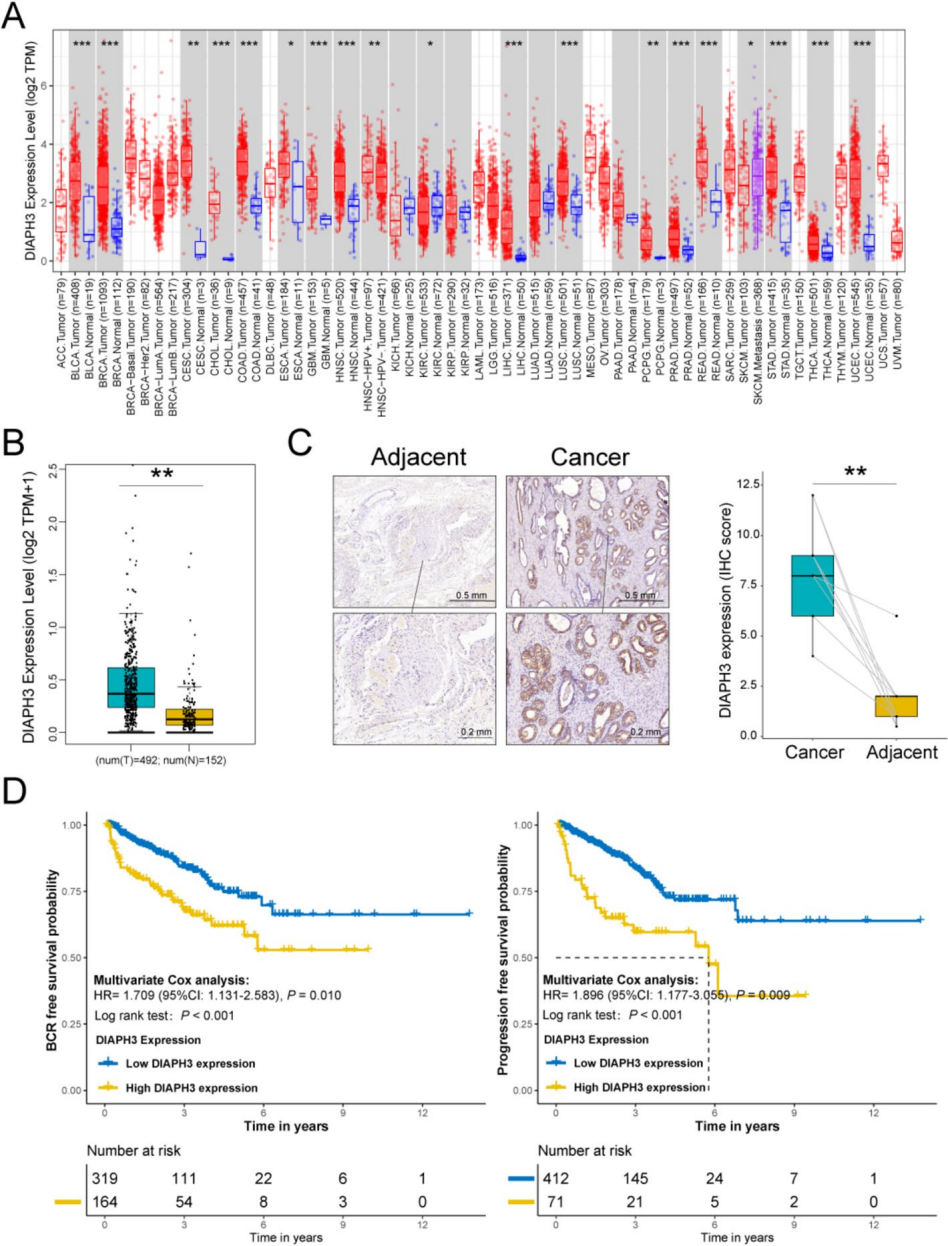
DIAPH3 expression and its prognostic value in TCGA cohort. **A** Pan-cancer analysis of DIAPH3 expression in TIMER2.0 database. **B** DIAPH3 expression in PCa and adjacent tissues of patients from GEPIA2 database, and normal prostate tissues were matched for GTEx database. **C** DIAPH3 expression in PCa and adjacent tissues from patients in Yinchuan cohort. **D** Association between DIAPH3 expression and BCRFS/PFS of TCGA PCa patients

The expression analysis of DIAPH3 in adjacent and cancer tissues was conducted using TCGA and Yinchuan cohorts. As shown in Fig. 1A, DIAPH3 expression was found to be upregulated in multiple cancers, including prostate cancer (PCa). When compared to normal prostate tissues from the GTEx database (Fig. 1B), elevated DIAPH3 expression was observed in cancer tissues. IHC results further confirmed that DIAPH3 expression was significantly higher in PCa tissues compared to paired adjacent normal tissues (Fig. 1C).

The prognostic value of DIAPH3 expression was evaluated with respect to biochemical recurrence-free survival (BCRFS) and progression-free survival (PFS) in the TCGA cohort. Kaplan–Meier curves indicated that patients with high DIAPH3 expression had poorer BCRFS and PFS compared to those with low DIAPH3 expression. Multivariate Cox regression, adjusted for age, TNM stage, Gleason score, PSA value, and surgical margin status, revealed that high DIAPH3 expression was an independent predictor of poor BCRFS (HR = 1.709, 95% CI: 1.131–2.583, P = 0.010) and PFS (HR = 1.896, 95% CI: 1.177–3.055, P = 0.009) (Fig. 1D).

**Fig. 2:**
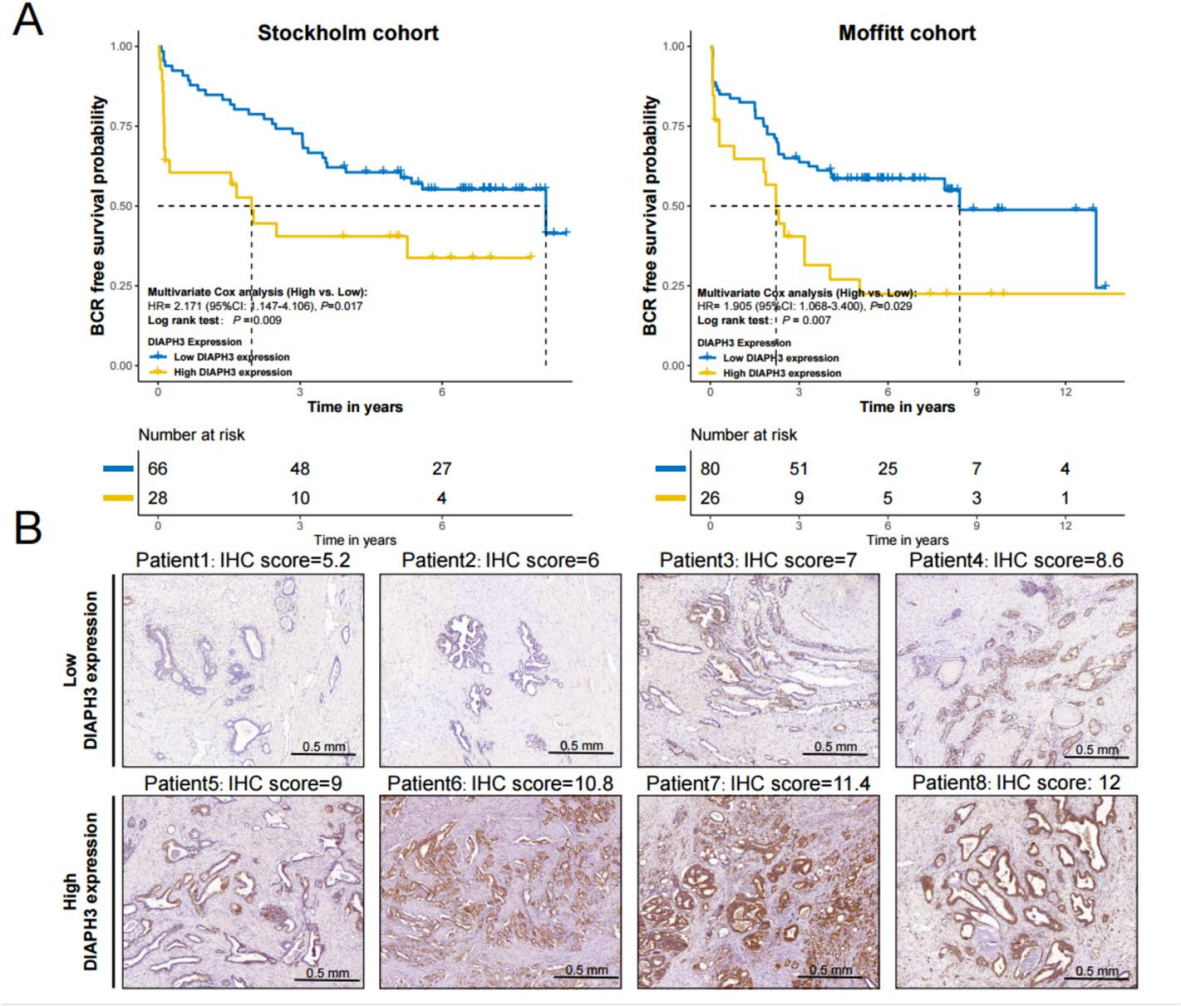
The association between DIAPH3 expression and prognosis of PCa patients with radical surgery in multiple datasets. **A** Association between DIAPH3 expression and BCRFS of PCa patients with radical surgery in Stockholm and Moffitt cohorts. **B** DIAPH3 expression in Yinchuan cohort patients with different progression status

To further validate these findings, we analyzed the effect of DIAPH3 expression on BCRFS in the Stockholm cohort (GSE70769), and Moffitt cohort (GSE54460) [23, 24]. As illustrated in Fig. 2A, high DIAPH3 expression was associated with worse BCRFS in both cohorts, with HRs of 2.171 (95% CI: 1.147–4.106, P = 0.017), 1.905 (95% CI: 1.068–3.400, P = 0.029).

Furthermore, we observed that DIAPH3 expression was significantly higher in patients with T3/T4 stage tumors compared to T2 stage tumors (Figure S3B, Figure S5), suggesting that elevated DIAPH3 expression is associated with more advanced tumor progression. These findings highlight the potential of DIAPH3 as a prognostic factor in PCa, correlating with both tumor progression and poorer clinical outcomes.

### Development and validation of prediction model for BCRFS and PFS in PCa patients underwent radical prostatectomy

**Fig. 3:**
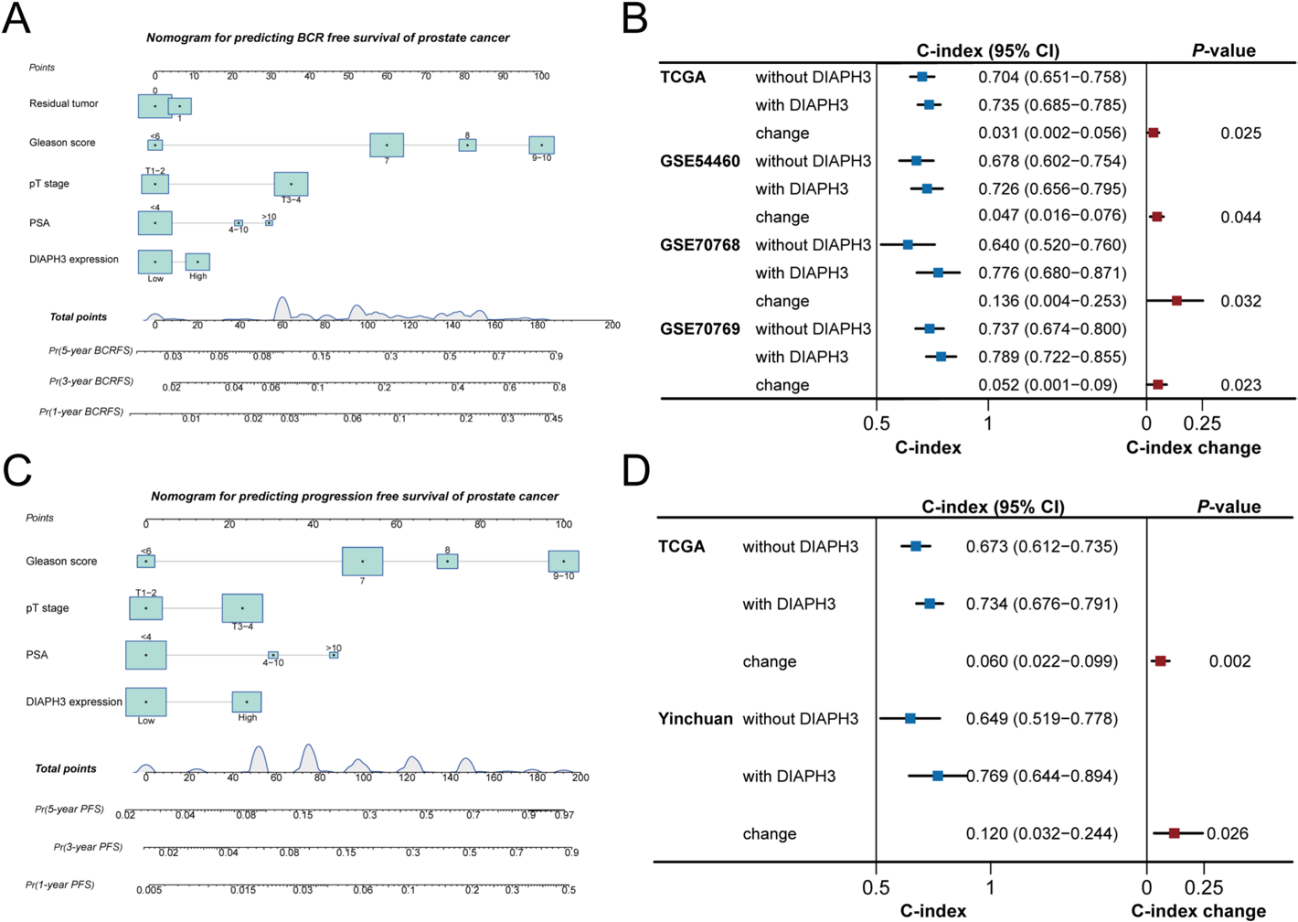
Development and validation of nomogram model predicting BCRFS and PFS for PCa after radical prostatectomy. **A** Nomogram model predicting BCRFS of PCa. **B** C-index of nomogram model and model without DIAPH3 for predicting BCRFS. **C** Nomogram model predicting PFS of PCa. **D** C-index of nomogram model and model without DIAPH3 for predicting PFS. Due to limitation of follow up time, the 5-year calibration curve in Yinchuan cohort was not calculated

In TCGA cohort, we performed stepwise regression analysis based on AIC value for BCRFS, and the results suggested the optimal model containing DIAPH3 expression, T stage, PSA value Gleason score and surgical margin status. The lowest AIC value was obtained for the final model, as well as the regression coefficients (β), p-values, and 95% confidence intervals (CI) for each selected variable are detailed in Figure S3 C. Therefore, we developed a nomogram model using above-mentioned variables to predict 1-year, 3-year and 5-year BCRFS (Fig. 3A). The nomogram model was internally validated in the TCGA cohort and independently validated in CamCap, Stockholm and Moffitt cohorts. As exhibited in Fig. 3B, the C-index result indicated that the nomogram model had an understanding predictive discrimination in four cohorts (C-index > 0.6). Notably, compared with model without DIAPH3, nomogram significantly improved C-index in four cohorts. Moreover, the calibration curves of 1-, 3- and 5-year BCRFS revealed a good calibrated ability of nomogram (Figure S1).

We employed the stepwise regression analysis based on AIC value for PFS, and the results suggested the optimal model containing DIAPH3 expression, T stage, PSA value and Gleason score. Therefore, a nomogram was developed using above-mentioned variables (Fig. 3C) to predict 1-year, 3-year and 5-year PFS. The nomogram model was internally validated in TCGA cohort and independently validated in Yinchuan cohort. As indicated in Fig. 3D, the C-index result suggested that nomogram model had an understanding predictive discrimination in the TCGA and Yinchuan cohorts (C-index > 0.6). Notably, compared with model without DIAPH3, the nomogram significantly improved C-index in TCGA and Yinchuan cohorts. Moreover, the calibration curves of 1-, 3- and 5-year PFS (the calibration curves of 5-year PFS in Yinchuan cohort was not calculated due to limitation of follow-up time) revealed a good calibrated ability of nomogram (Figure S2).

### High DIAPH3 expression correlates with improved BCRFS and PFS in RRT-treated patients

**Fig. 4:**
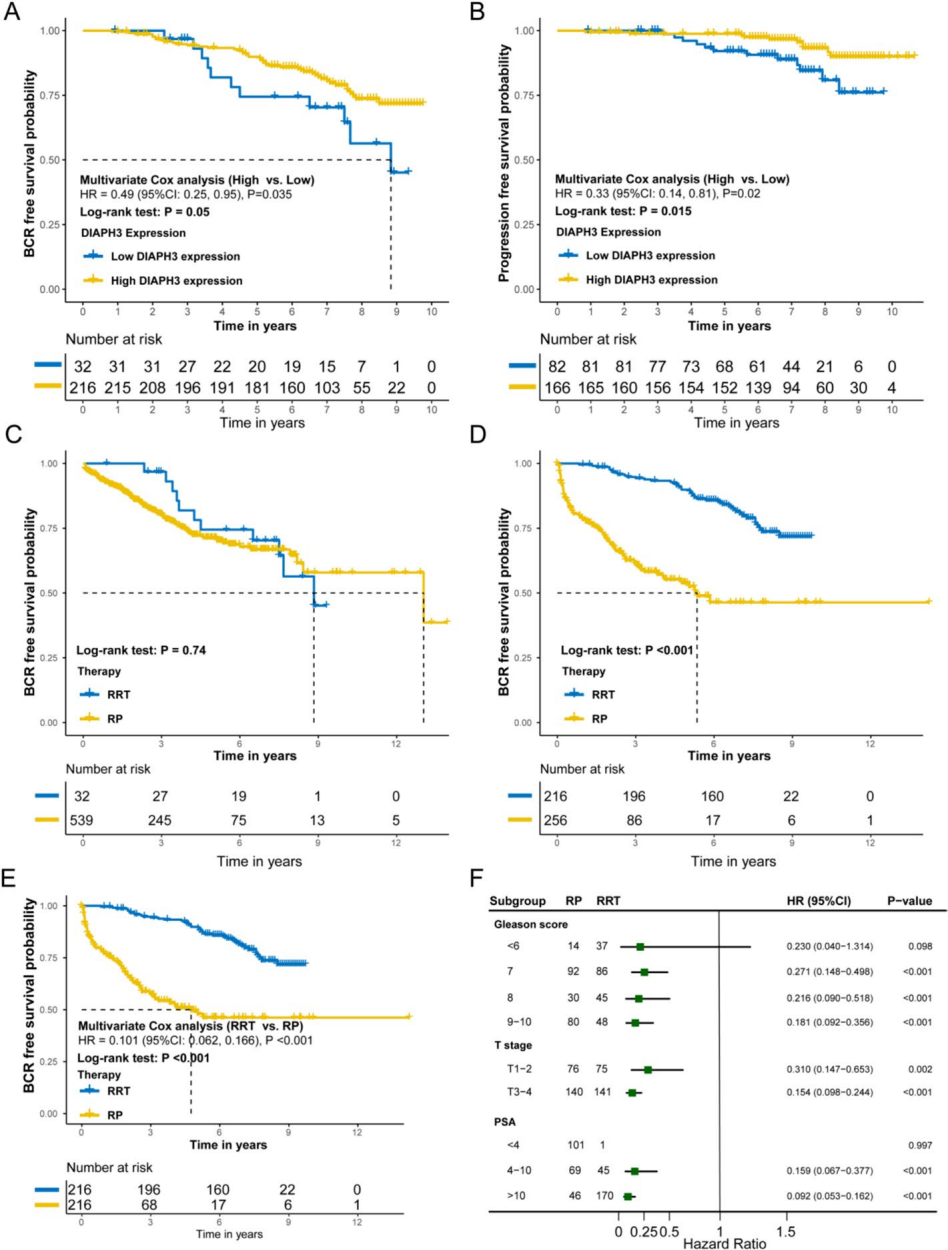
The association between DIAPH3 expression and prognosis of PCa patients with radical radiotherapy. **A** Association between DIAPH3 expression and BCRFS of PCa patients with radical radiotherapy in UK cohort. **B** Association between DIAPH3 expression and PFS of PCa patients with radical radiotherapy in UK cohort. **C** BCRFS of PCa patients of integrated cohorts with radical radiotherapy and radical surgery in low DIAPH3 expression group. **D** BCRFS of PCa patients of integrated cohorts with radical radiotherapy and radical surgery in high DIAPH3 expression group. **E** BCRFS of PCa patients with radical radiotherapy and radical surgery in high DIAPH3 expression group after propensity score matching. **F** Subgroup analysis of BCRFS of PCa patients with radical radiotherapy and radical surgery in high DIAPH3 expression group after propensity score matching.(RP is the reference, Patients with high DIAPH3 results may benefit more from RRT)

In the UK cohort, multivariate analysis adjusting for age, Gleason score, PSA value, and T stage revealed that high DIAPH3 expression was associated with improved BCRFS (HR = 0.49, 95% CI: 0.25–0.95, *P* = 0.035) and PFS (HR = 0.33, 95% CI: 0.14–0.81, *P* = 0.02) in prostate cancer patients treated with RRT (Fig. 4A, B).

Furthermore, we integrated the CamCap, Stockholm, Moffitt, and UK cohorts to compare BCRFS between patients undergoing RP and RRT. Interestingly, among patients with low DIAPH3 expression, no significant difference in KM curves was observed between RP and RRT (Fig. 4C). However, a significant difference was found between RP and RRT in patients with high DIAPH3 expression (Fig. 4D). After propensity score matching (1:1), multivariate Cox analysis revealed that the HR for RRT versus RP was 0.101 in patients with high DIAPH3 expression (95% CI: 0.062–0.166, *P* < 0.001; Fig. 4E). Subgroup analysis of the matched data further confirmed significant differences between RP and RRT across multiple subgroups of patients with high DIAPH3 expression (Fig. 4F).

### DIAPH3 expression was associated with tumor immune microenvironment

**Fig. 5:**
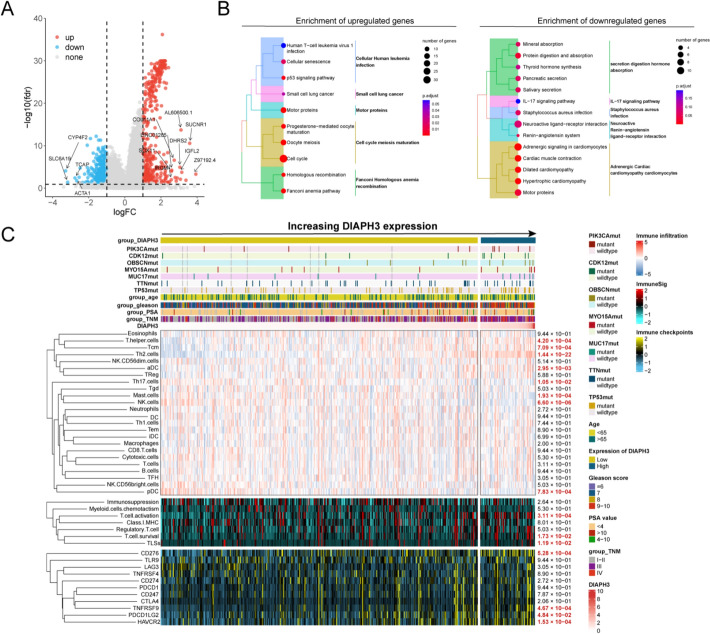
Exploration of potential biological process, somatic mutation and immune microenvironment status related to DIAPH3 expression. **A** Differential expressed genes between patients with low and high DIAPH3. **B** Gene function enrichment analysis for identified differential expressed genes based on KEGG annotation. **C** Somatic mutation and tumor immune microenvironment status between patients with low and high DIAPH3 expression

After the identification of DIAPH3 as a poor variable in PCa prognosis, we further analyzed the potential biological process related to DIAPH3 expression. We first performed the DEG analysis between high and low DIAPH3 expression groups, and CYP4F2, SLC6A19, TCAP, ACTA1 were found a down-regulation in high DIAPH3 expression group while COL11A1, SUCNR1, SOX11, IGFL2, IRGM and DHRS2 were found a up-regulation in high DIAPH3 expression group (Fig. 5A). Gene function enrichment analysis was performed to explore the DIAPH3-related pathways based on KEGG gene sets, and clustered results suggested the involvement of DIAPH3 in cell cycle regulation and IL-17 pathway (Fig. 5B).

Furthermore, we explored the effect of DIAPH3 on tumor somatic mutation and tumor immune microenvironment (Fig. 5C). In the high DIAPH3 expression group, PIK3CA, CDK12, OBSCN, MYO15A, MUC17, TTN and TP53 mutated more frequently than in the low DIAPH3 expression group. In tumor immune microenvironment, Th2 cell was significantly enriched in high DIAPH3 expression group while NK cell and pDC (plasmacytoid dendritic cell) were lack in these patients. Specifically, DIAPH3 upregulation is associated with increased CD80/CD28 (T-cell activation markers) and reduced CD274/PD-L1 and CD276/B7-H3 (immune checkpoint molecules) across multiple cancer types (Figure S3A).

### Low DIAPH3 expression predicts enhanced immunotherapy sensitivity in a pan-cancer cohort

We comprehensively investigated the expression pattern, prognostic value, and immunotherapy relevance of DIAPH3 across cancers. The following analysis of immunotherapy response, however, was conducted and is interpreted in a pan-cancer context. As shown in Figure S6 A, DIAPH3 expression was significantly upregulated in the majority of cancers compared with normal tissues. Survival analyses indicated that high DIAPH3 expression served as a risky prognostic factor in multiple cancer types, including LIHC, PAAD, and STAD, while external validations in DKFZ2018, GSE21034, and GSE70769 datasets consistently confirmed its association with poor outcomes (HR > 1, P < 0.05, Figure S6B, D) Furthermore, univariate logistic regression demonstrated that DIAPH3 overexpression was strongly associated with cancer risk, particularly in PAAD (OR = 72.75, Figure S6 C).

**Fig. 6:**
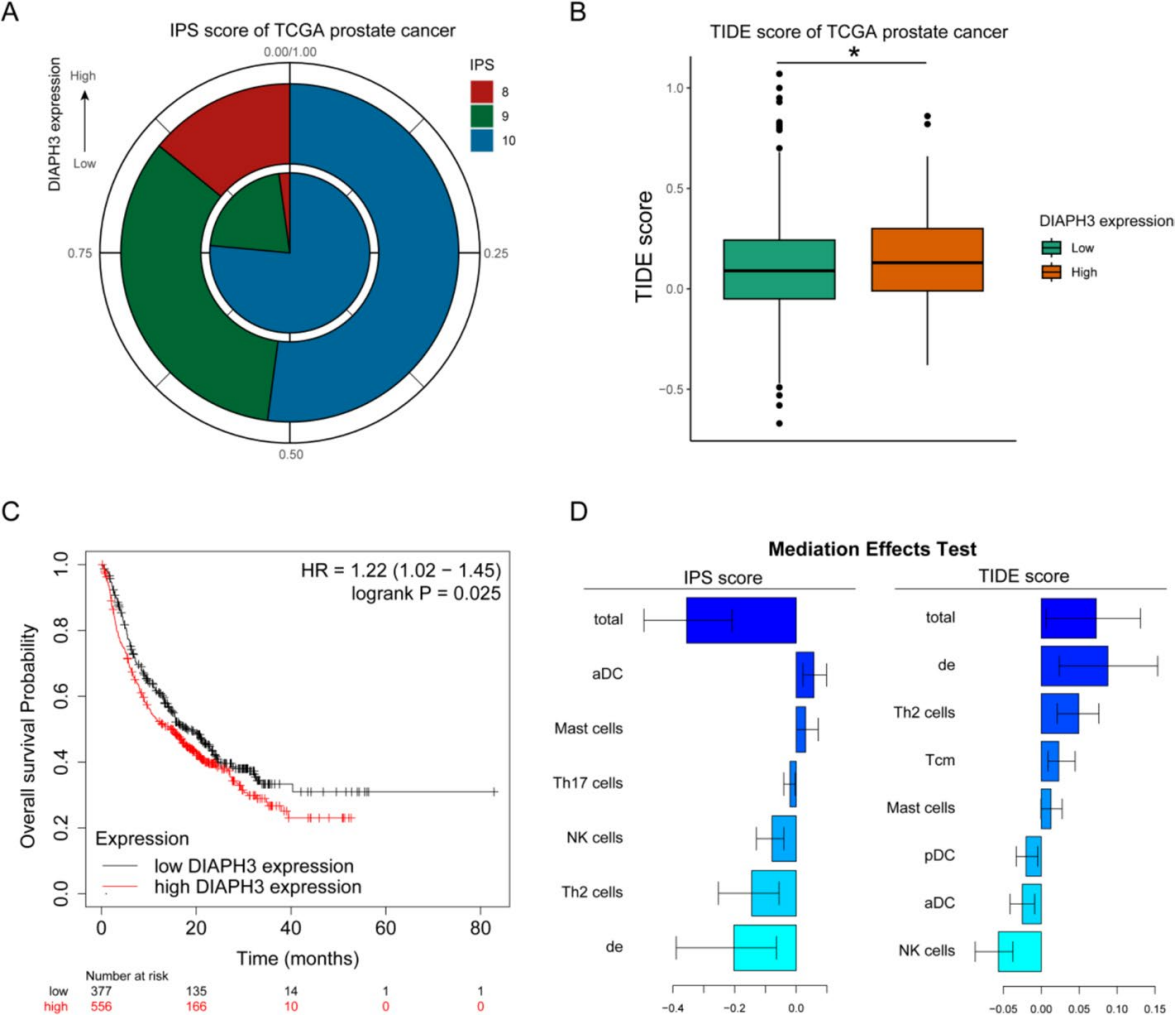
The association between DIAPH3 expression and immunotherapy response in a pan-cancer cohort. **A** IPS in TCGA-PRAD patients with low and high DIAPH3 expression. **B** TIDE score in TCGA-PRAD patients with low and high DIAPH3 expression. **C** Association between DIAPH3 expression and overall survival of pan-cancer receiving immunotherapy, including bladder cancer, esophageal adenocarcinoma, glioblastoma, melanoma and urothelial cancers and so on. **D** Mediation analysis of immune cells in effect of DIAPH3 on immunotherapy reponse. *Note: The analysis and significant finding presented here are based on the integrated pan-cancer cohort

To further assess its potential role in immunotherapy response, we utilized TIDE and IPS scores. Patients with low DIAPH3 expression exhibited significantly higher IPS scores and lower TIDE scores, suggesting that this group may be more sensitive to immune checkpoint blockade therapies than those with high DIAPH3 expression. Specifically, in Fig. 6A, B, the IPS inner circle represents the proportion of IPS scores in the DIAPH3 low-expression group, whereas the outer circle represents that of the high-expression group. The survival analysis in Fig. 6C was derived from a pan-cancer cohort (n = 933) to validated the effect of DIAPH3 expression on immunotherapy including PD-1, PD-L1 and CTLA4 in the DIAPH3 high and low expression group. There was a significant difference in overall survival between low and high DIAPH3 expression groups (Log-rank P-value = 0.025), and the HR of high DIAPH3 expression vs. low DIAPH3 expression was 1.22 (95%CI: 1.02–1.45). To specifically address the relevance of DIAPH3 in prostate cancer immunotherapy, we performed a subgroup analysis of the prostate cancer patients (n = 87) within this cohort. In this PCa-specific analysis, the association between DIAPH3 expression and overall survival after immunotherapy did not reach statistical significance (HR = 1.32, 95% CI: 0.89–1.96, log-rank P = 0.17). Therefore, the significant predictive value for immunotherapy response is primarily derived from the pan-cancer context. The potential role of DIAPH3 in prostate cancer immunotherapy remains to be confirmed in larger, prospective studies. Due to importance of immune microenvironment in immunotherapy response, we analyzed the potential mediation effect of immune microenvironment between DIAPH3 and immunotherapy response. As shown in Fig. 6D, Th2 cell was found to be most important mediators among 24 immune cells.

### Potential drug interventions for DIAPH3

**Fig. 7:**
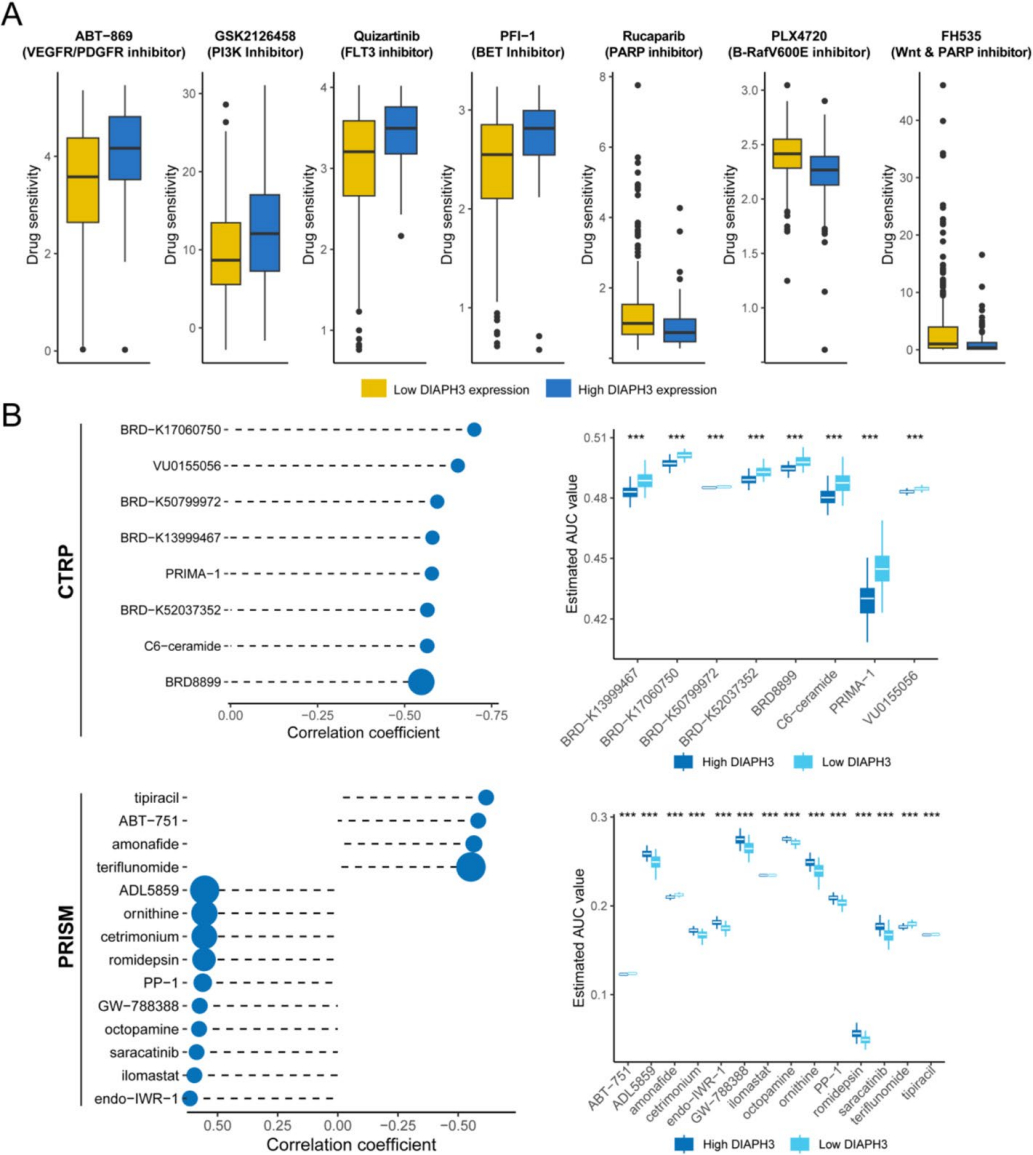
Identification of potential drug interventions for DIAPH3 expression. **A** Exploratory analysis of DIAPH3 expression and inferred drug sensitivity based on GDSC database. The box plots illustrate the distribution of predicted IC50 values. These results represent computational predictions that require further statistical and experimental validation. **B** DIAPH3 expression and inferred drug sensitivity based on CTRP and PRISM databases

Using the Oncopredict algorithm, we performed an exploratory analysis to investigate potential associations between DIAPH3 expression and sensitivity to various anti-cancer agents. This computational screening identified several compounds for which sensitivity appeared to differ between DIAPH3-high and DIAPH3-low tumors. Specifically, the model suggested that tumors with low DIAPH3 expression might exhibit increased sensitivity to ABT − 869, GSK2126458, Quizartinib, and PFI − 1. Conversely, tumors with high DIAPH3 expression might show greater sensitivity to Rucaparib, PLX4720, and FH535 (Fig. 7A). While these patterns are intriguing and point to potential therapeutic vulnerabilities, this analysis remains hypothesis-generating. The statistical significance of these differences could not be robustly confirmed in the current dataset, and thus these findings require future validation in independent cohorts or functional experiments. These findings suggest that DIAPH3 expression could serve as a biomarker for guiding personalized therapy selection, with different treatment strategies indicated for DIAPH3-low versus DIAPH3-high tumors. Consistent with this notion, analysis of compounds from the CTRP and PRISM databases further identified several agents, including tipiracil, AB-751, amonafide, and teriflunomide, to which patients with high DIAPH3 expression demonstrated enhanced sensitivity (Fig. 7B). This reinforces the concept that DIAPH3 expression is linked to specific therapeutic vulnerabilities. It is important to emphasize that the drug associations identified here are based on computational predictions from cell line databases. While these findings highlight potential therapeutic vulnerabilities linked to DIAPH3 expression, they do not constitute direct evidence of clinical efficacy in prostate cancer patients. The identified compounds should be considered as promising candidates for further experimental validation in prostate cancer models and subsequent clinical investigation.

## Discussion

In this study, we identified DIAPH3 as a potential risk factor for recurrence events (BCRFS and PFS) in PCa patients. We developed and validated nomogram models to predict 1-, 3-, and 5-year BCRFS and PFS. Notably, DIAPH3 expression was significantly correlated with conventional clinical prognostic factors such as Gleason score, PSA level, and T stage (Figure S5), which may partly explain its prognostic value. Additionally, we found that DIAPH3 may be involved in the regulation of cell cycle and tumor immune microenvironment. Analysis of a pan-cancer immunotherapy cohort indicated that low DIAPH3 expression was associated with a more favorable response to immunotherapy across multiple cancer types. While this suggests a potential immunomodulatory role, its specific applicability to prostate cancer warrants future investigation.

DIAPH3 has been reported as an oncogene in multiple cancers, including pancreatic cancer [9], cervical cancer [25], lung cancer [26] and hepatocellular cancer [27]. In these malignancies, DIAPH3 may promote tumor cell proliferation and migration through mechanisms involving mTOR and selenoprotein TrxR1-mediated antioxidant effects. However, DIAPH3 may exert different effect on other cancer types. In triple-negative breast cancer, DIAPH3 can suppress the migration and invasion by downregulating the expression of Rho-guanosine-5’-triphosphate [11]. Therefore, the prognostic effect of DIAPH3 expression in cancer patients may vary across cancer types. In our study, utilizing follow-up data from the TCGA and Yinchuan cohorts, we identified that high DIAPH3 expression constitutes a risk factor of PFS in PCa patients. We also found that DIAPH3 expression is elevated in the T3/T4 progression stage compared to the T2 stage, which implies that DIAPH3 expression accelerates disease progression in prostate cancer (PCa). We also found that higher DIAPH3 expression was associated with higher TNM stage, higher PSA level, and higher Gleason score.

The prognosis of PCa is strongly associated with tumor stage, Gleason score and PSA levels [28]. Emerging evidences indicated that certain genes hold significant potential as biomarkers for evaluating the prognosis of PCa patients [29]. However, clinicians lack a quantified tool to effectively take use of these factors. Nomogram, which integrated multi-factors and provided a straightforward visual presentation of predictions, has been employed for prognosis evaluation in various cancers [30, 31]. Therefore, we employed stepwise regression to optimize the included factors into model and integrated these factors to construct a nomogram. Our drug sensitivity analysis provides a crucial layer to the prognostic value of DIAPH3. Importantly, the relationship between DIAPH3 and drug response is not monolithic. We found that low DIAPH3 expression defines a tumor subset with vulnerability to agents like PI3K and BET inhibitors, whereas high DIAPH3 expression is associated with sensitivity to PARP inhibitors. This apparent paradox underscores the multifaceted role of DIAPH3 in cancer biology and resolves the seemingly contradictory statements by demonstrating that DIAPH3 expression does not simply predict ‘better’ or ‘worse’ drug response, but rather a ‘different’ response profile. These findings position DIAPH3 not only as a prognostic biomarker but also as a potential companion diagnostic for personalized treatment strategy in PCa, suggesting that therapeutic decisions could be optimized based on DIAPH3 expression status. Notably, DIAPH3 has been previously reported as a biomarker for drug sensitivity in previous study, including MEK inhibitor and taxanes [12, 32]. Inhibiting DIAPH3 expression may enhance the sensitivity of MEK inhibitor and taxanes in PCa. In our study, high DIAPH3 expression was identified as a poor factor of BCRFS and PFS after radical surgery, but a favor factor of BCRFS and PFS after radical radiotherapy. The debate regarding the selection between RP and RRT for PCa patients has been a subject of considerable academic interest [33, 34]. Although our study suggested that PCa patients with high DIAPH3 expression may be more suitable for RRT than RP, the conclusion required further validation through prospective studies.

Previous research has demonstrated an association between DIAPH3 and tumor immune cell infiltration in a pan-cancer analysis, suggesting a relationship between DIAPH3 expression and B cells, effector T cells, and macrophage infiltration [13]. In the context of pancreatic cancer, significant correlations were observed between DIAPH3 expression and T helper 2 (Th2) cell infiltration, pDC infiltration and myeloid-derived suppressor cell infiltration [14]. Consistent with findings in pancreatic cancer, our study reveals that higher DIAPH3 expression in PCa is associated with increased Th2 cell infiltration and decreased NK and pDC cell infiltration. Th2 cell infiltration was recognized as a recurrence marker in PCa patients [35]. As highlighted in previous studies, tumor immune microenvironment was an important factor of cancer recurrence and metastasis [18, 36–38]. Additionally, our findings also suggests that Th2 cell may be a significant factor influencing the impact of DIAPH3 on immunotherapy. Current research supports that Th2 cells serve as an immunosuppressive signal that enhance the function of M2 macrophages, thus promoting tumor growth and immunotherapy resistance [39]. Up to now, the role of DIAPH3 in regulating the tumor immune microenvironment remains unclear. Previous study has proposed that extracellular vesicles may mediate the interaction between DIAPH3 and tumor microenvironment [40]. Our analysis indicated the IL-17 pathway, a crucial factor in promoting PCa progression and metastasis [41, 42], may represent an additional mechanism through another way of immune modulation of DIAPH3.

However, our study still has several limitations. First, we did not delve deeply into the regulatory mechanisms governing DIAPH3 expression. Second, our findings lack validation in large-scale clinical cohorts. Third, we did not further investigate the specific role of DIAPH3 in modulating the IL-17 signaling pathway.Further research is required to elucidate the relationship between DIAPH3 and tumor immune environment as well as its involvement in PCa immunotherapy. In addition, the nomogram should be validated in larger cohorts to ensure its reliability.

## Supplementary Information

Additional file1

Additional file2

Additional file3

Additional file4

Additional file5

Additional file6

## Data Availability

The datasets used during analyses in the current study are available in the TCGA database (https://portal.gdc.cancer.gov/) and GEO database (https://www.ncbi.nlm.nih.gov/geo). The information of patients in Yinchuan cohort can be required from corresponding author.

## Abbreviations

PSA: Prostate-specific antigen
DIAPH3: Diaphanous-related formin 3
GEO: Gene Expression Omnibus
BCRFS: Biochemical recurrence-free survival
PFS: Progression free survival
RP: Radical prostatectomy
RRT: Radical radiotherapy
KM: Kaplan–meier
AIC: Akaike information criterion
BH: Benjamini-Hochberg
FDR: False discovery rate
KEGG: Kyoto Encyclopedia of Genes and Genomes
ssGSEA: Single-sample gene set enrichment analysis
IPS: Immunophenoscore
TIDE: Tumor immune dysfunction and exclusion
pDC: Plasmacytoid dendritic cell
