# Supplementary figures and images for: DIAPH3 is a multifaceted prognostic biomarker that links immunotherapy response to tumor microenvironment in prostate cancer
Source: Discov Oncol. 2026 Jan 16;17:286. doi: 10.1007/s12672-026-04413-6 (PMC12891325; doi:10.1007/s12672-026-04413-6)

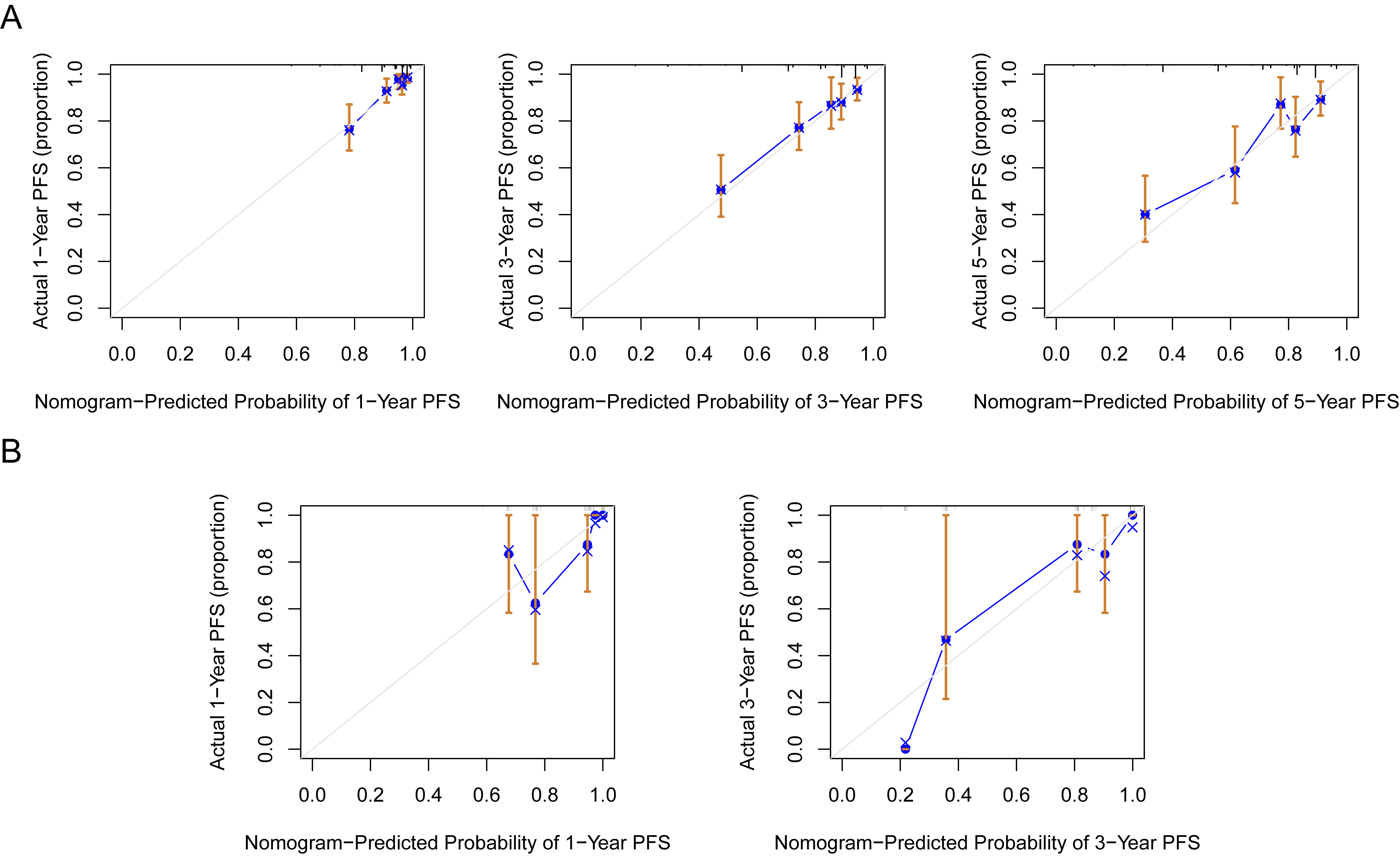

Supplement: Supplementary file 2 — Additional file2 [file 12672_2026_4413_MOESM2_ESM.tif]

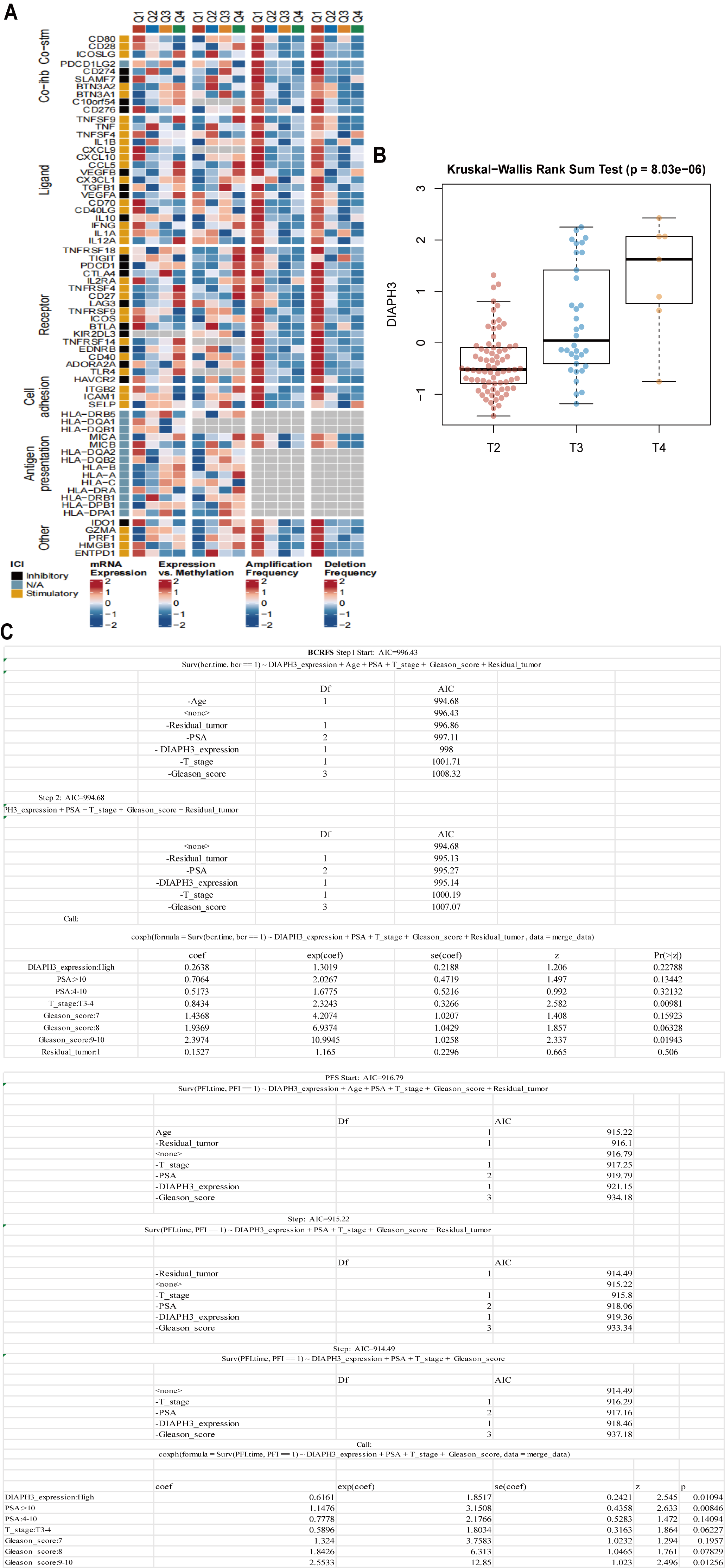

Supplement: Supplementary file 3 — Additional file3 [file 12672_2026_4413_MOESM3_ESM.tif]

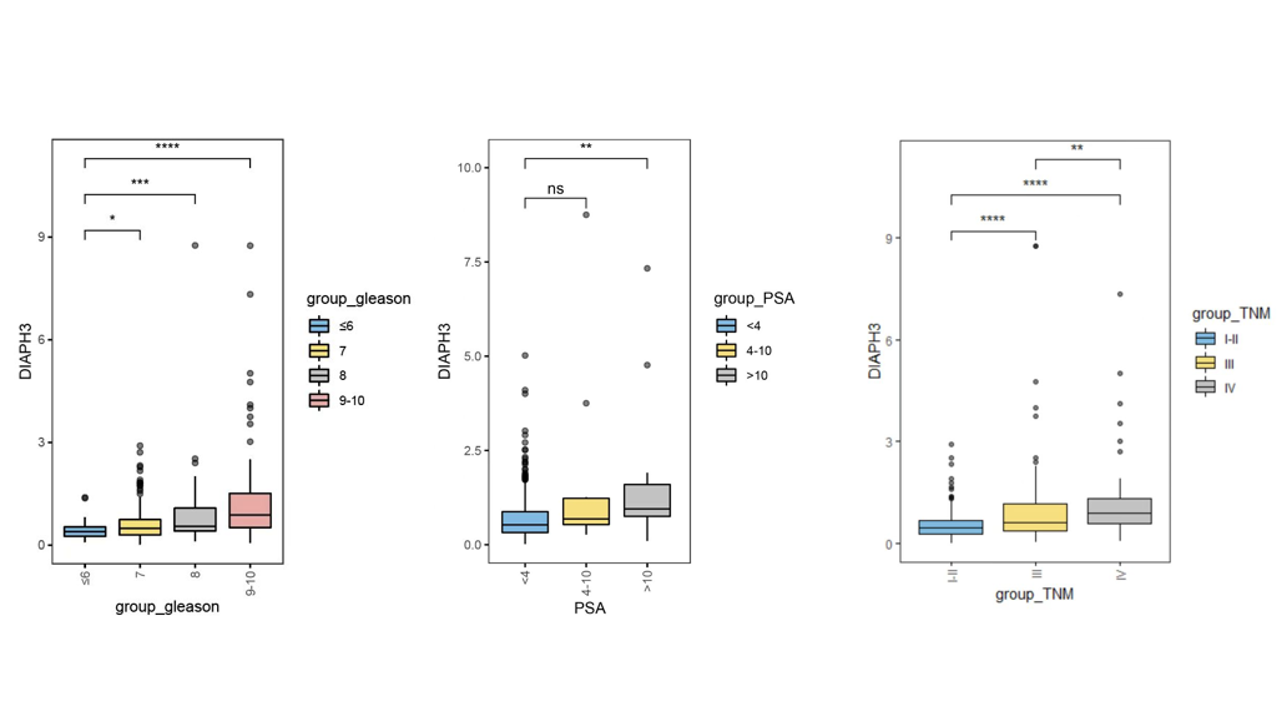

Supplement: Supplementary file 5 — Additional file5 [file 12672_2026_4413_MOESM5_ESM.tif]

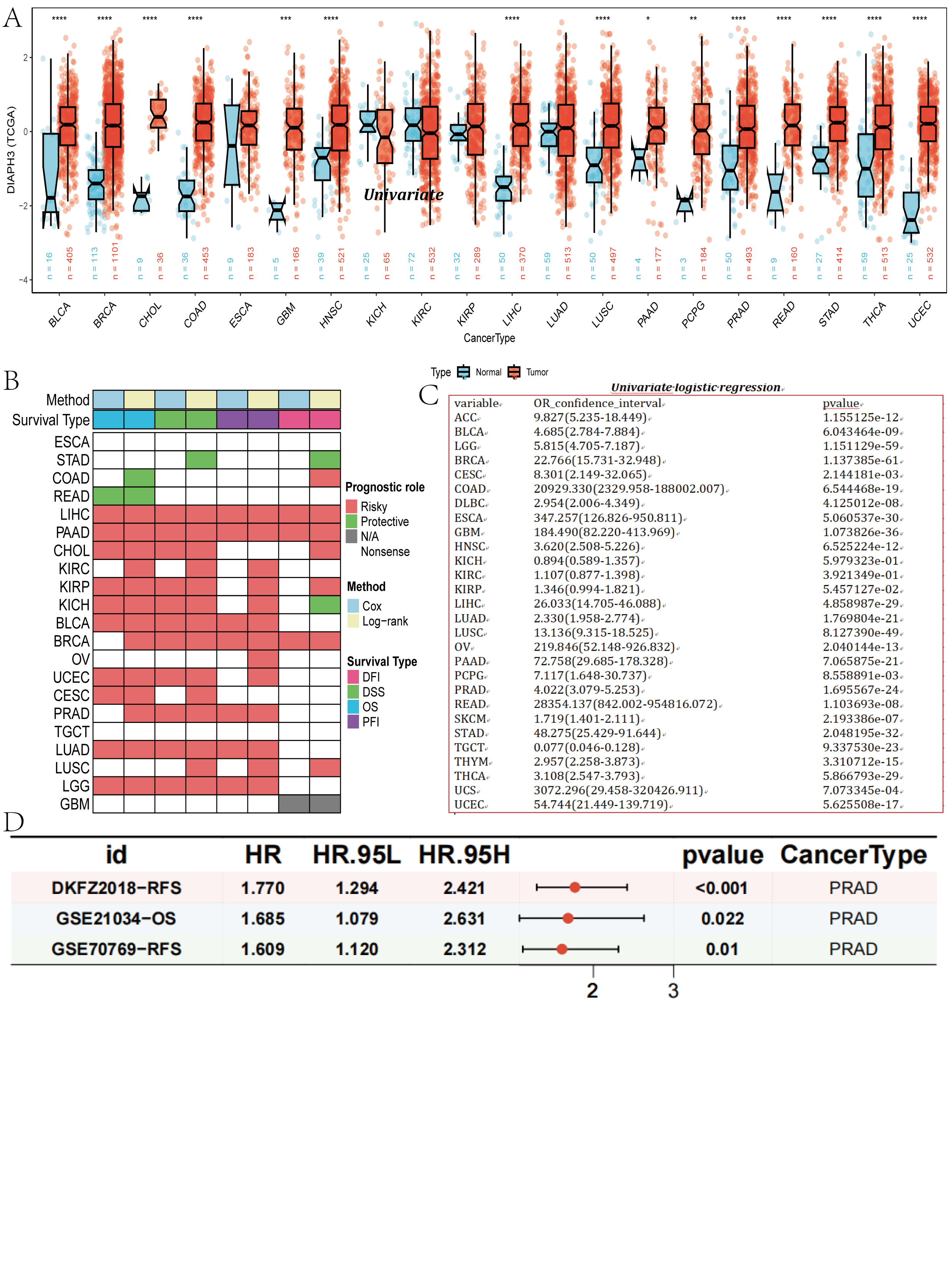

Supplement: Supplementary file 6 — Additional file6 [file 12672_2026_4413_MOESM6_ESM.tif]
