# Supplementary material for: DIAPH3 is a multifaceted prognostic biomarker that links immunotherapy response to tumor microenvironment in prostate cancer
Source: Discov Oncol. 2026 Jan 16;17:286. doi: 10.1007/s12672-026-04413-6 (PMC12891325; doi:10.1007/s12672-026-04413-6)
Supplement: Supplementary file 4 — Additional file4 [file 12672_2026_4413_MOESM4_ESM.pdf]

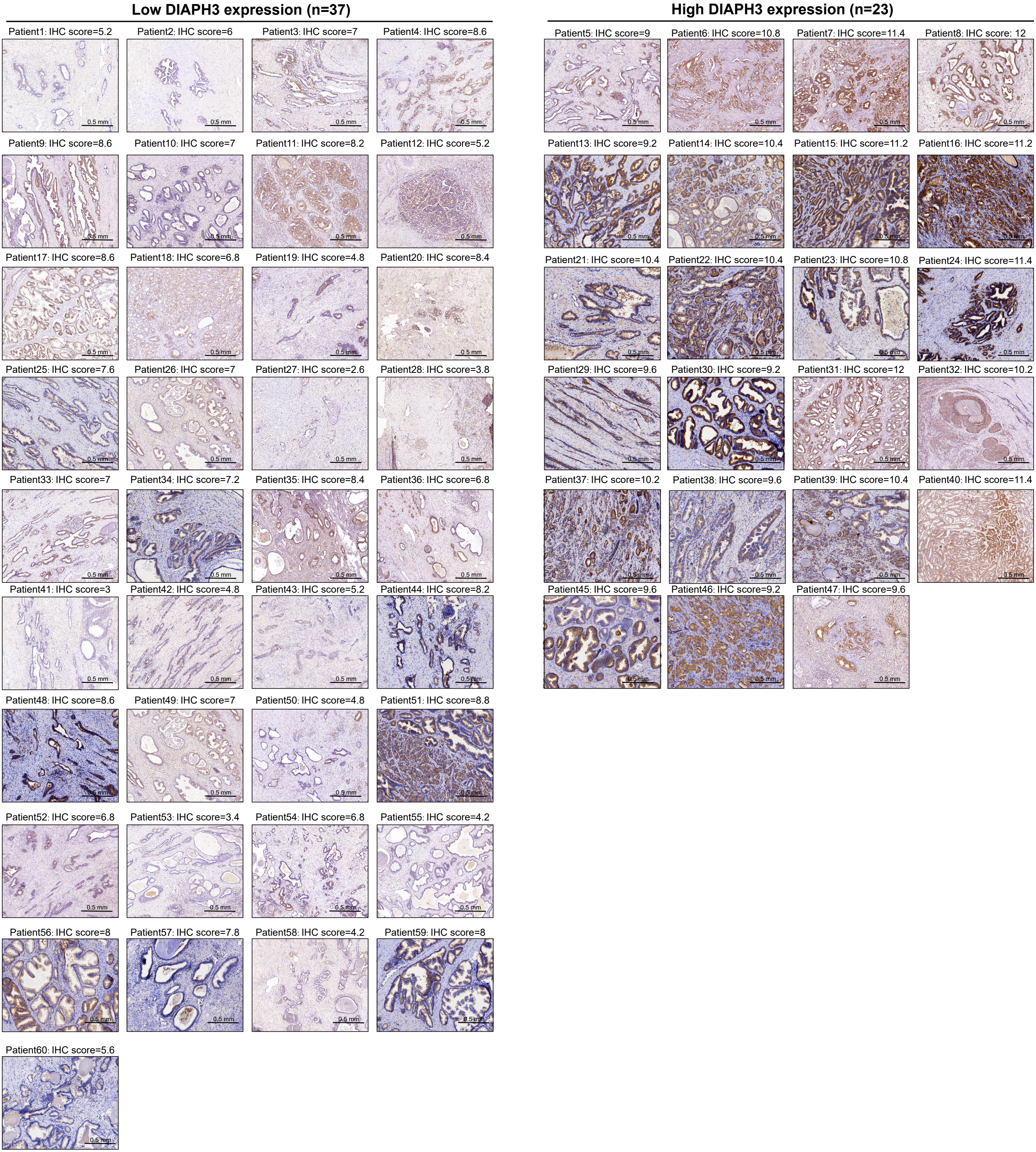
Low DIAPH3 expression (n=37)
High DIAPH3 expression (n=23)
Patient1: IHC score=5.2
Patient2: IHC score=6
Patient3: IHC score=7
Patient4: IHC score=8.6
Patient5: IHC score=9
Patient6: IHC score=10.8
Patient7: IHC score=11.4
Patient8: IHC score: 12
Patient9: IHC score=8.6
Patient10: IHC score=7
Patient11: IHC score=8.2
Patient12: IHC score=5.2
Patient13: IHC score=9.2
Patient14: IHC score=10.4
Patient15: IHC score=11.2
Patient16: IHC score=11.2
Patient17: IHC score=8.6
Patient18: IHC score=6.8
Patient19: IHC score=4.8
Patient20: IHC score=8.4
Patient21: IHC score=10.4
Patient22: IHC score=10.4
Patient23: IHC score=10.8
Patient24: IHC score=11.4
Patient25: IHC score=7.6
Patient26: IHC score=7
Patient27: IHC score=2.6
Patient28: IHC score=3.8
Patient29: IHC score=9.6
Patient30: IHC score=9.2
Patient31: IHC score=12
Patient32: IHC score=10.2
Patient33: IHC score=7
Patient34: IHC score=7.2
Patient35: IHC score=8.4
Patient36: IHC score=6.8
Patient37: IHC score=10.2
Patient38: IHC score=9.6
Patient39: IHC score=10.4
Patient40: IHC score=11.4
Patient41: IHC score=3
Patient42: IHC score=4.8
Patient43: IHC score=5.2
Patient44: IHC score=8.2
Patient45: IHC score=9.6
Patient46: IHC score=9.2
Patient47: IHC score=9.6
Patient48: IHC score=8.6
Patient49: IHC score=7
Patient50: IHC score=4.8
Patient51: IHC score=8.8
Patient52: IHC score=6.8
Patient53: IHC score=3.4
Patient54: IHC score=6.8
Patient55: IHC score=4.2
Patient56: IHC score=8
Patient57: IHC score=7.8
Patient58: IHC score=4.2
Patient59: IHC score=8
Patient60: IHC score=5.6
